# Supplementary material for: Integrated Transcriptome and Metabolome Analysis Reveals Phenylpropanoid Biosynthesis and Phytohormone Signaling Contribute to “Candidatus Liberibacter asiaticus” Accumulation in Citrus Fruit Piths (Fluffy Albedo)
Source: Int J Mol Sci. 2022 Dec 9;23(24):15648. doi: 10.3390/ijms232415648 (PMC9779719; doi:10.3390/ijms232415648)
Supplement: Supplementary file 1 [file ijms-23-15648-s001.zip › Tables S1-S6.pdf]

Table S1. Sequencing data and quality of RNA-seq.

Sample	Raw_reads	Clean_reads	Clean_bases	Error_rate	Q20	Q30
DF1	46079072	44706334	6.71G	0.03	97.57	93.18
DF2	46723390	45332678	6.80G	0.03	97.64	93.33
DF3	50614798	49297276	7.39G	0.03	97.69	93.45
DL1	42161150	40657838	6.10G	0.03	97.67	93.37
DL2	43173672	41948482	6.29G	0.03	97.71	93.51
DL3	42649176	41503620	6.23G	0.03	97.87	93.81
HF1	46986566	45082956	6.76G	0.03	97.77	93.67
HF2	47740160	46782602	7.02G	0.03	97.84	93.81
HF3	45319374	43999042	6.60G	0.03	97.79	93.59
HL1	44151220	43091706	6.46G	0.03	97.6	93.05
HL2	47171146	45969718	6.90G	0.03	97.79	93.52
HL3	47316370	45758626	6.86G	0.03	97.82	93.63

Note: DF, Diseased fruit pith. DL, Diseased leaf. HF, Healthy fruit pith. HL, Healthy leaf. Numbers following the sample names indicate replication code.

Table S2. Summary of genome mapping results using RNA-seq data of each sample.

Sample	Total_reads	Total_map	Unique_map
DF1	44706334	42219808(94.44%)	41131693(92.00%)
DF2	45332678	42510065(93.77%)	41371682(91.26%)
DF3	49297276	46516589(94.36%)	45254927(91.80%)
DL1	40657838	38480723(94.65%)	37335476(91.83%)
DL2	41948482	39688367(94.61%)	38459510(91.68%)
DL3	41503620	39503819(95.18%)	38351002(92.40%)
HF1	45082956	42215392(93.64%)	41074328(91.11%)
HF2	46782602	44149651(94.37%)	42945811(91.80%)
HF3	43999042	41619402(94.59%)	40522875(92.10%)
HL1	43091706	40934515(94.99%)	39765720(92.28%)
HL2	45969718	43317602(94.23%)	42092417(91.57%)
HL3	45758626	43311193(94.65%)	42056422(91.91%)

Note: DF, Diseased fruit pith. DL, Diseased leaf. HF, Healthy fruit pith. HL, Healthy leaf. Numbers following the sample names indicate replication code.

Table S3. Eighty-eight differentially accumulated metabolites with fold change > 5 in the fruits.

ID	Name	FC	log_2FC	VIP
Com_105_neg	IBA	3894.81	11.93	2.91
Com_933_neg	Acetaminophen glucuronide	320.76	8.33	2.86
Com_150_pos	Scopoletin	236.91	7.89	2.87
Com_2677_pos	6-methyl-5-(2-methyl-1,3-thiazol-4-yl)-2-(phenylthio)nicotinonitrile	161.68	7.34	2.84
Com_2579_pos	Scutellarin	105.98	6.73	2.95
Com_6514_neg	Dactylorhin E	79.41	6.31	2.77
Com_9518_pos	Kanamycin	68.00	6.09	2.27
Com_2512_pos	Sitagliptin	64.87	6.02	2.41
Com_3496_pos	4-(3,4-dihydro-2H-1,5-benzodioxepin-7-ylamino)-4-oxobutanoic acid	63.74	5.99	2.80
Com_418_neg	Plantagoside	61.46	5.94	2.72
Com_6770_pos	Paliperidone	52.44	5.71	2.37
Com_1579_pos	Coixol	46.73	5.55	2.27
Com_3902_neg	Tricin 5-O-hexosyl-O-hexoside	33.32	5.06	2.96
Com_6635_neg	4,5-DCQA Isochlorogenic acid C	32.54	5.02	2.56
Com_20187_neg	16 -Hydroxyestrone	32.30	5.01	2.93
Com_2648_pos	4-phenoxyphenyl 4-hydroxypiperidine-1-carboxylate	31.53	4.98	2.70
Com_8195_neg	Cephalotaxine	31.50	4.98	2.27
Com_2255_neg	Esculin	30.97	4.95	2.86
Com_5273_pos	6-Formyl-isoophiopogonanone A	26.49	4.73	2.83
Com_6004_neg	2'-O-acetylpoliumoside	24.74	4.63	2.52
Com_13099_neg	Pinoresinol Diglucoside	23.98	4.58	2.39
Com_2322_pos	1-Methylguanosine	22.69	4.50	2.11
Com_12908_neg	Heteroclitin D	22.48	4.49	2.25
Com_10762_neg	Baohuoside I	21.38	4.42	2.41
Com_2092_pos	2-{[2-(3-cyano-2-pyridinyl)hydrazino]carbonyl}benzoic acid	20.90	4.39	2.40
Com_2850_pos	10-Deacetylasperulosidic acid	20.03	4.32	2.62
Com_1579_neg	4-Acetyl-3-hydroxy-5-methylphenyl 3-D-glucopyranoside	19.28	4.27	1.99
Com_1142_neg	Chlortetracycline	18.40	4.20	2.25
Com_2217_pos	N-Feruloyltyramine	18.29	4.19	2.55
Com_3195_neg	Catharanthine hemitartrate	18.27	4.19	2.33
Com_1813_pos	RNH	17.46	4.13	2.34
Com_3843_pos	Thiamine	17.24	4.11	2.60
Com_3399_pos	Valepotriate	16.81	4.07	2.11
Com_1610_pos	Glu-Gln	15.74	3.98	1.76

Com_7916_neg	L-Dopa	15.44	3.95	2.58
Com_2274_neg	Yangonin	14.79	3.89	1.85
Com_11698_neg	Gomisin D	14.70	3.88	2.21
Com_88_pos	N-Feruloyl putrescine	14.10	3.82	2.39
Com_89_pos	3,4-Methylenedioxy-汐-<br>pyrrolidinopropiophenone	14.05	3.81	2.39
Com_13116_pos	Morphine	13.11	3.71	2.80
Com_7145_pos	Thromboxane B2	12.60	3.66	2.08
Com_4404_neg	Curculigoside	12.06	3.59	2.30
Com_18151_neg	Rosavin	11.65	3.54	2.72
Com_11443_pos	2-(cyclopropylcarbonyl)-3-<br>(4-fluoroanilino)acrylonitrile	11.53	3.53	1.94
Com_1949_pos	Ligustilide	11.37	3.51	2.14
Com_11225_neg	Guan-fu base A	11.28	3.50	2.66
Com_1819_neg	3',4',5'-Tricetin 5-O-hexoside	11.24	3.49	2.35
Com_10903_neg	Esculetin	11.19	3.48	2.50
Com_2792_neg	5-O-Methylvisammioside	10.94	3.45	2.24
Com_7129_pos	2,2-dimethyl-6,7-di[(4-nitrobenzyl)oxy]<br>chroman-4-one	10.73	3.42	1.70
Com_4499_pos	Tricin 5-O-3-D-glucoside	10.41	3.38	2.00
Com_25099_neg	beta-Nicotinamide Mononucleotide	10.37	3.37	2.72
Com_15076_neg	Citrinin	10.20	3.35	2.45
Com_2789_pos	7-hydroxy-6-methoxy-2H-chromen-2-one	10.20	3.35	2.66
Com_7435_neg	Limocitrin O-hexoside	9.95	3.31	2.28
Com_18018_neg	Hederacoside D	9.77	3.29	2.33
Com_305_pos	Gentisic acid	9.59	3.26	1.68
Com_14592_pos	DLK	9.44	3.24	1.93
Com_11341_pos	1,8-diphenoxy-9,10-dihydroanthracene-<br>9,10-dione	9.29	3.22	2.67
Com_2979_pos	Phenylglyoxylic acid	9.27	3.21	2.40
Com_10177_neg	3-O-p-coumaroyl quinic acid O-hexoside	8.74	3.13	2.40
Com_6351_neg	Epimedin A	8.69	3.12	2.60
Com_8954_neg	Benzyl 6-O-beta-D-glucopyranosyl-<br>beta-D-glucopyranoside	8.38	3.07	2.56
Com_5352_neg	Eleutheroside E	8.36	3.06	1.88
Com_5847_neg	Tenuifoliside B	8.28	3.05	2.57
Com_4257_neg	D(-)-Amygdalin	8.18	3.03	1.75
Com_23858_neg	Chelidonine	8.02	3.00	2.13
Com_23122_pos	Ingenol-3-angelate	7.83	2.97	2.49
Com_25830_neg	Raceanisodamine	7.71	2.95	2.87
Com_9377_neg	6''-o-acetylgenistin	7.57	2.92	2.13
Com_4151_pos	3-(dimethylamino)-1-<br>(5-methyl-3-phenyl-4-isoxazolyl)-<br>2-propen-1-one	7.36	2.88	1.82

Com_7549_neg	TBHQ	7.31	2.87	1.84
Com_23083_pos	N-Feruloylspermidine	7.03	2.81	2.57
Com_14463_pos	Levodopa	6.88	2.78	1.88
Com_29795_neg	1-(4-fluorophenyl)-2-(4-methoxyphenyl)-4-(2-naphthyl)butane-1,4-dione	6.48	2.70	2.20
Com_7478_neg	Astringin	6.46	2.69	1.66
Com_7654_pos	Acetaminophen	6.24	2.64	2.17
Com_19666_pos	Ginkgolide C	5.98	2.58	2.41
Com_26237_neg	Deacetyltaxol	5.89	2.56	2.65
Com_5450_pos	3-N-butyl-4,5-dihydrophthalide	5.87	2.55	2.43
Com_15957_pos	Phe-Pro	5.84	2.55	2.01
Com_1794_pos	Arctiin	5.70	2.51	1.52
Com_31328_neg	7-O-Methylaloeresin A	5.31	2.41	2.84
Com_8218_neg	1-[6-(benzyloxy)-3-(tert-butyl)-2-hydroxyphenyl]ethan-1-one	5.26	2.39	1.79
Com_4962_neg	1-O-Feruloyl quinic acid	5.25	2.39	1.83
Com_25406_neg	Mulberroside A	5.20	2.38	2.13
Com_13783_neg	N4-(3,4-dimethylphenyl)quinazolin-4-amine hydrochloride	5.07	2.34	2.31
Com_11253_neg	Miyabenol C	5.01	2.32	2.34

Note: FC, Fold change. The metabolites with VIP > 1 and p-value < 0.05 and FC ⩾ 2 or FC ⩽ 0.5 were considered to be differential metabolites in the study.

Table S4. Nine differentially accumulated metabolites with fold change > 5 in the leaves.

ID	Name	FC	log2FC	VIP
Com_2857_neg	Chlorogenic acid	11.79	3.56	2.58
Com_12908_neg	Heteroclitin D	11.00	3.46	2.14
Com_8082_neg	Procyanidin B2	6.10	2.61	2.62
Com_2992_pos	4-(pentyloxy)benzene-1-carbohydrazide	5.92	2.57	2.14
Com_1301_pos	diethyl 2-[(4-benzhydrylpiperidino) methylidene]malonate	5.90	2.56	1.58
Com_3296_pos	3-(3,4,5-trimethoxyphenyl) propanoic acid	5.75	2.52	2.92
Com_1933_pos	2-morpholinophenyl 1-methyl-1H-imidazole-4-sulfonate	5.69	2.51	1.59
Com_7031_neg	5-O-p-Coumaroyl shikimic acid	5.16	2.37	1.73
Com_5964_pos	Corynoline	5.14	2.36	2.09

Note: FC, Fold change. The metabolites with VIP > 1 and p-value < 0.05 and FC ⩾ 2 or FC ⩽ 0.5 were considered to be differential metabolites in the study.

Table S5. Quality indicators of total RNA samples for RNA-seq.

Sample	Concentration (ng/ul)	Total (ul)	Quantum (ug)	Integrity Value	Test Conclusions
DF1	45	40	1.80	7.2	A
DF2	69	40	2.76	8.1	A
DF3	31	40	1.24	7.7	A
DL1	195	40	7.80	8.5	A
DL2	107	40	4.28	6.4	A
DL3	382	40	15.28	8.5	A
HF1	38	40	1.52	6.9	A
HF2	174	35	6.09	9.1	A
HF3	19	40	0.76	8.6	A
HL1	624	40	24.96	7.2	A
HL2	368	40	14.72	6.1	A
HL3	450	40	18.00	6.0	A

Note: DF, Diseased fruit pith. DL, Diseased leaf. HF, Healthy fruit pith. HL, Healthy leaf. Numbers following the sample names indicate replication code.

Table S6. Quality controls of the liquid chromatography-tandem mass spectrometry quantitative analysis of plant hormones.

Phytohormone	Abbreviations and modes		CAS No	Chemical formula	Retention time	Equation	Degree of fitting
Indole-3-acetic acid	IAA	-	87-51-4	$C_{10}H_9NO_2$	3.84	Y = 2.413 e5X	0.9991
Gibberellin A1	GA1	-	545-97-1	$C_{19}H_{24}O_6$	3.39	Y = 1.406 e6X	0.9994
Gibberellin A3	GA3	-	1977-6-5	$C_{19}H_{22}O_6$	3.35	Y = 6.602 e5X	0.9993
Gibberellin A4	GA4	-	468-44-0	$C_{19}H_{24}O_5$	4.64	Y = 1.481 e6X	0.9994
Gibberellin A7	GA7	-	510-75-8	$C_{19}H_{22}O_5$	4.59	Y =	0.9993

						2.329e6X	
Jasmonic acid	JA	-	3572-66-5	$C_{12}H_{18}O_3$	4.41	Y = 1.44e6X	0.9989
Jasmonoyl-isoleucine	JA-Ile	-	120330-93-0	$C_{18}H_{29}NO_4$	4.73	Y = 2.249e6X	0.9999
Salicylic acid	SA	-	69-72-7	$C_7H_6O_3$	3.17	Y = 5.526e5X	0.9993
Abscisic acid	ABA	-	14375-45-2	$C_{15}H_{20}O_4$	4.05	Y = 5.313e5X	0.9996
1-aminocyclopropanecarboxylic acid	ACC	+	22059-21-8	$C_4H_7NO_2$	0.41	Y = 3.98e5X	0.9984
Trans-zeatin	TZ	+	1637-39-4	$C_{10}H_{13}N_5O$	1.06	Y = 1.108e6X	0.9989
N6-(Δ2-isopentenyl)adenine	NIA	+	2365-40-4	$C_{10}H_{13}N_5$	3.52	Y = 1.932e6X	0.9992
N6-(Δ2-isopentenyl)adenosine	NIAS	+	7724-76-7	$C_{15}H_{21}N_5O_4$	3.69	Y = 9.621e5X	0.9906
Brassinolide	BR	+	72962-43-7	$C_{28}H_{48}O_6$	5	Y = 9.899e4X	0.9977
methyl jasmonate	MJA	+	1211-29-6	$C_{13}H_{20}O_3$	5.19	Y = 7.766e3X	0.9983
Trans-zeatin-riboside	TZR	+	6025-53-2	$C_{15}H_{21}N_5O_5$	2.38	Y = 7.152e5X	0.9994
