# Supplementary figures and images for: Integrated Transcriptome and Metabolome Analysis Reveals Phenylpropanoid Biosynthesis and Phytohormone Signaling Contribute to “Candidatus Liberibacter asiaticus” Accumulation in Citrus Fruit Piths (Fluffy Albedo)
Source: Int J Mol Sci. 2022 Dec 9;23(24):15648. doi: 10.3390/ijms232415648 (PMC9779719; doi:10.3390/ijms232415648)

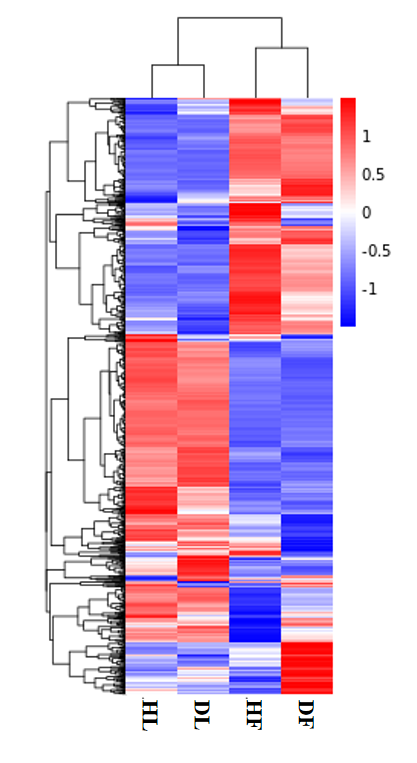

Supplement: Supplementary file 1 [file ijms-23-15648-s001.zip › Figure S1.tif]

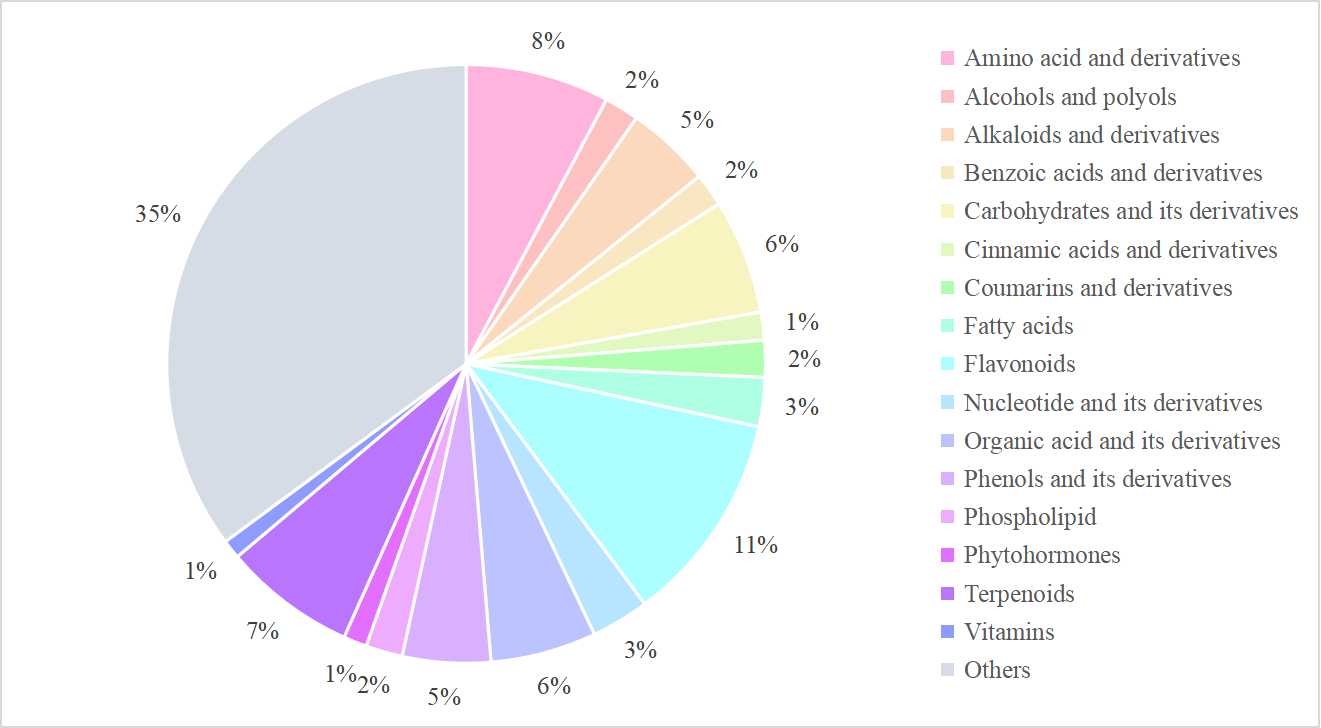

Supplement: Supplementary file 1 [file ijms-23-15648-s001.zip › Figure S2.jpg]

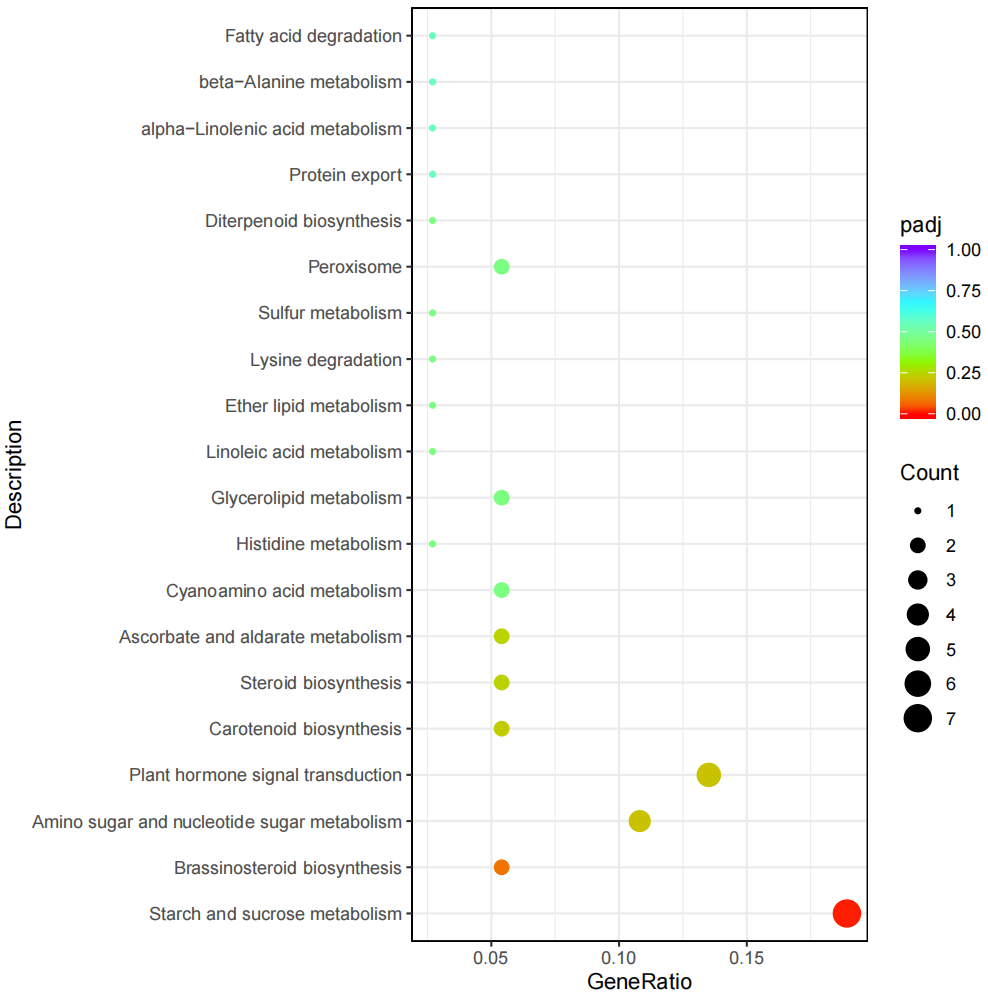

Supplement: Supplementary file 1 [file ijms-23-15648-s001.zip › Figure S3.tif]

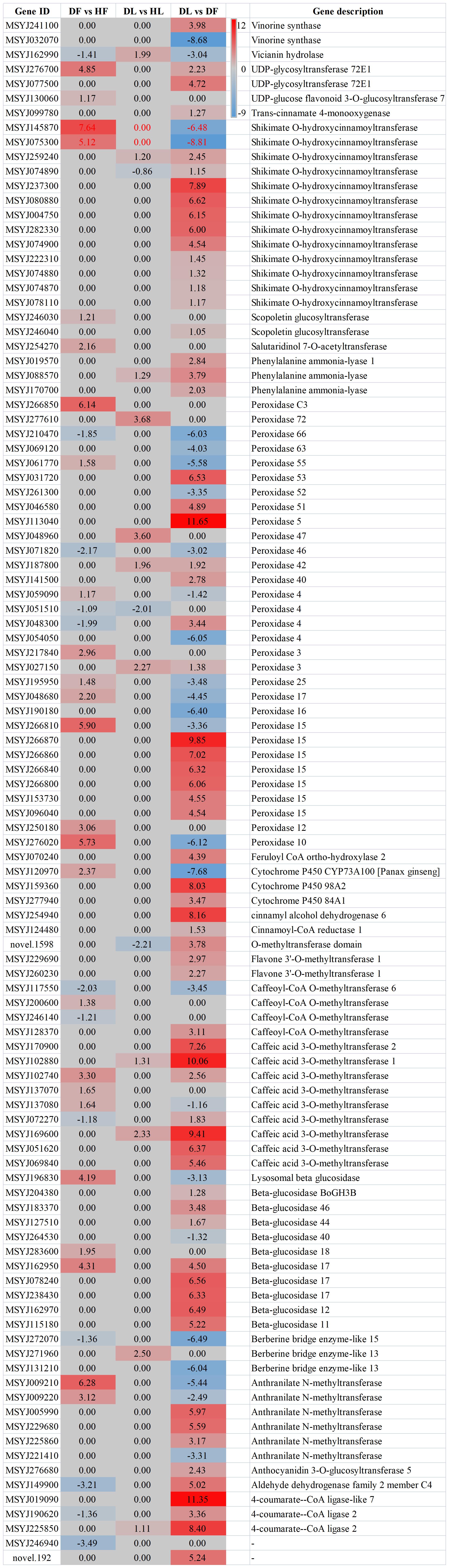

Supplement: Supplementary file 1 [file ijms-23-15648-s001.zip › Figure S4.jpg]
